# Coupling transcriptional and post-transcriptional miRNA regulation in the control of cell fate

**DOI:** 10.18632/aging.100085

**Published:** 2009-09-08

**Authors:** Reut Shalgi, Ran Brosh, Moshe Oren, Yitzhak Pilpel, Varda Rotter

**Affiliations:** ^1^ Department of Molecular Cell Biology, Weizmann Institute of Science, Rehovot, Israel; ^2^ Department of Molecular Genetics, Weizmann Institute of Science, Rehovot, Israel

**Keywords:** miRNA, Dicer, cancer, genome integrity, senescence

## Abstract

miRNAs function as a critical regulatory layer in development, differentiation, and the maintenance of cell fate. Depletion of miRNAs from embryonic stem cells impairs their differentiation capacity. Total elimination of miRNAs leads to premature senescence in normal cells and tissues through activation of the DNA-damage checkpoint, whereas ablation of miRNAs in cancer cell lines results in an opposite effect, enhancing their tumorigenic potential. Here we compile evidence from the literature that point at miRNAs as key players in the maintenance of genomic integrity and proper cell fate. There is an apparent gap between our understanding of the subtle way by which miRNAs modulate protein levels, and their profound impact on cell fate. We propose that examining miRNAs in the context of the regulatory transcriptional and post-transcriptional networks they are embedded in may provide a broader view of their role in controlling cell fate.

## miRNAs are key regulators of cell fate

miRNAs have emerged in the past decade as important players in numerous cellular and organismal processes in animals and plants [1]. Deletion of the *Dicer *gene, encoding the critical enzyme involved in miRNA processing and maturation, is embryonic lethal in both mice [2] and zebrafish [3]. Accordingly, many studies showed, using conditional elimination of *Dicer*, that miRNAs are crucial for the proper spatiotemporal development of various tissues and organs ([2,4-9] and reviewed in [10]). Further, mouse embryonic stem (ES) cells defective in miRNA processing were shown to proliferate slower [11], and to be impaired in their ability to differentiate [8]. In parallel, other studies have shown a major role for miRNAs in development, indicating that many miRNAs are upregulated during the process of ES cell differentiation ([12] and reviewed in [13]). Many miRNAs also play a role in differentiation processes in the adult organism, including  hematopoiesis [14] and the germinal center response [15]. In fact, the first miRNAs to be discovered, *lin-4* and *let-7* in *C. elegans*, regulate epithelial cell differentiation [16,17]. In addition, manipulations of individual miRNA genes were shown to result in marked defects at the organismal level ([18,19] and reviewed in [20]). Based on these accumulated observations it is plausible to suggest that in many cases miRNAs are indeed a part of the driving force of differentiation processes. miRNAs were also shown to regulate many cellular processes [21,22], such as cell growth and proliferation (reviewed in [23,24]) and apoptosis (reviewed in [25]). It appears, therefore, that miRNAs are crucial players in the regulation and determination of cell fate.

## miRNAs - guardians of genome integrity?

Lu et al. [26] carried out an extensive analysis of miRNA expression in human cancer. This study, that included a global expression profiling of miRNAs across a large set of tumors, demonstrated that miRNA expression profiles can be used to classify human cancers of unknown origin. In addition, the researchers made the very interesting observation that, in general, tumors have lower levels of miRNAs than normal tissues. The authors suggested that the observed low global levels of miRNAs may be a reflection of the de-differentiated state of tumors.

An alternative, complementary explanation might be that tumors evolve to silence the miRNA pathway during the course of cancer progression. In other words, globally avoiding regulation of gene expression by miRNAs may be one of the many ways of cancer cells to enhance their proliferation and tumorigenic potential.

**Figure 1.**
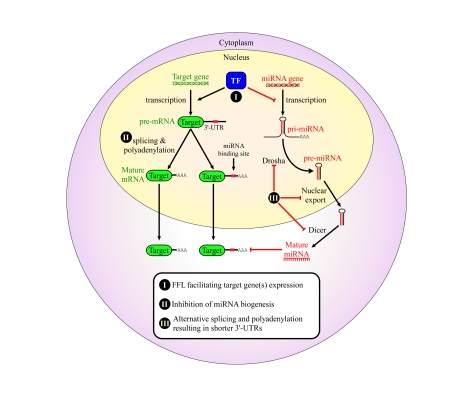
Proposed mechanisms for avoidance of regulation by miRNAs in cancer cells. We propose that cancers may evolve to avoid regulation by miRNAs in order to enhance their tumorigenic potential. This might occur through a variety of mechanisms: (I) combined transcriptional/post-transcriptional FFL wiring, which may enhance the repression of several co-regulated miRNAs, thereby facilitating the expression of the mutual target genes; (II) global avoidance of miRNA regulation via expression of shorter 3' UTRs [42]; (III) global reduction in miRNA levels by impairing miRNA biogenesis in various ways, some of which were shown to happen in tumors, such as inhibition of *Drosha *processing [39,40] and pre-miRNA nuclear retention [36]. All of these are suggested as means that developing tumors may evolve to enhance proliferation and increase genome instability.

Several lines of evidence support the idea that proliferating cells and cancer cells in particular, find many different ways to avoid post-transcriptional regulation by miRNAs (Figure 1). Some of these mechanisms are straightforward, and are in agreement with what we know of tumor suppressors and oncogenes. For example, the *MYC *oncogenic transcription factor (TF) was found in a lymphoma mouse model to mediate widespread repression of a large set of miRNAs, contributing to tumorigenesis [27]. Other mechanistic possibilities for tumors to avoid posttranscriptional regulation by miRNAs include epigenetic silencing, mutation and deletion of genomic loci encoding for miRNAs [28-33]. A prominent example is the *miR-15a/16-1 *cluster, residing in the *DLEU2 *non-coding RNA, which was long known to be frequently deleted in leukemia [34,35], and was later shown to harbor these miRNAs [29]. Another newly described mechanism is the interruption of the miRNA biogenesis pathway, by processes such as nuclear retention of unprocessed pre-miRNAs [36], or pri- and pre-miRNA processing blockage such as in the case of inhibition of maturation of the *let-7 *family by the *Lin28 *protein [37-39]. *Lin28* was further shown to promote cancer, and this was attributed to its repression of the *let-7 *miRNA family [40]. A recent report implicates *p53* in the enhancement of miRNA maturation for many miRNAs following DNA damage [41], attesting to global miRNA upregulation as a possible anti-cancer mechanism. Additional highly intriguing phenomenon was reported by Sandberg et al. [42], indicating that proliferating cells tend to employ alternative polyadenylation or alternative splicing in order to express mRNAs with shorter 3' UTRs, having fewer miRNA binding sites. These shorter mRNAs avoid post-transcriptional regulation by miRNAs, thus potentially enhancing their protein level. This phenomenon represents another path by which proliferating cells achieve the same goal - avoiding miRNA-mediated silencing, presumably in order to accelerate proliferation.

The most striking evidence in support of the 'miRNA avoidance' strategy played by tumors is shown by two seemingly contradictory studies, one focusing on cancer cells and the other on normal cells. The study by Kumar et al. [43] reported that the ablation of miRNAs in various cancer cell lines resulted in enhanced cellular transformation, evident by increased colony formation efficiency *in vitro* and increased tumor burden *in vivo*. On the other hand, Mudhasani et al. [44] showed that the total elimination of miRNAs using conditional *Dicer *knock-out results in premature senescence in normal mouse embryonic fibroblasts (MEFs). This effect was also apparent at the level of the organism, as the knock-out of*Dicer* in keratinocytes and skin epidermis of adult mice resulted in senescence-induced hairloss and skin aging [44].

At first glance, these two studies seem to disagree. How is it possible that a similar manipulation would enhance proliferation in one system, and cause a proliferation arrest or senescence in the other? A potential solution to this conflict would consider that the same event can lead to two opposite outcomes, depending on the cellular context. For example, activation of an oncogene, such as *RAS*, is one of the hallmarks of cancer, and when occurring in cancer cells will cause the enhancement of their tumorigenic phenotype. However, in normal cells, oncogene activation will often lead to genomic instability, which is sensed by the DNA damage checkpoint, and leads to *p53* and *ARF*-dependent senescence, a phenomenon known as "oncogene-induced senescence" [45]. Importantly, the phenomenon described by Mudhasani et al. [44] was not a classical case of oncogene-induced senescence, as it was not accompanied by the upregulation of the oncogenes *MYC *or *RAS*, (two well known activators of oncogene-induced senescence), even though they are documented miRNA targets [46-48]. Interestingly, however, the depletion of miRNAs led to DNA damage, as evident by γ*H2A.X* staining, and consequently, through activation of the *p19ARF* and *p53*-dependent DNA-damage checkpoint, resulted in premature senescence.

Therefore, in this case too, the same event of global miRNA depletion induced the DNA damage checkpoint in normal cells due to proper *p19ARF* and *p53* activation, while in cancer cells it led to enhanced transformation, where these checkpoint response pathways are frequently inactivated, and genomic instability enhances tumorigenesis [49].

Importantly, as we outline here, inactivation of miRNA-mediated silencing is not only capable in principle of influencing cell fate, following genetic manipulations as shown by Mudhasani et al. and Kumar et al. [43,44]*, *but may actually occur* in vivo *during tumorigenesis [26,42]. It therefore seems likely that miRNAs are not only necessary for proliferation and differentiation in normal cells, but also act to maintain normal cell proliferation, and may be thought of as "guardians" of genome integrity. In cancer cells, on the other hand, inactivation of the miRNA-mediated silencing pathway and the avoidance of miRNA regulation contribute to transformation (Figure 1). In principle we can therefore consider miRNAs as a regulatory barrier whose removal may be part of a series of events that ultimately lead to cancer.

## A conceptual gap between the influence of miRNAs on protein levels and their effects on cell fate

miRNAs can exert their silencing effects by cleavage of their target mRNAs and by inhibition of their translation. A common knowledge in the field was that animal miRNAs exert most of their silencing through the inhibition of translation, rather than through the degradation of their targets, and that this was due to a low overall degree of sequence complementarity that animal miRNAs share with their target sites on 3' UTRs of mRNAs [1]. In fact, the first discovered miRNAs in *C. elegans*, *lin-4*, was shown to inhibit the translation of its target *Lin-14*, without affecting its mRNA levels [50,51]. Mechanistically, it became evident that the miRNA-effector protein complex, the *RISC*, is enzymatically capable of both mRNA cleavage and inhibition of translation [52,53]. Lim et al. then showed that miRNAs can influence the mRNA levels of their target genes [54]. Using overexpression of miRNAs followed by global expression profiling using microarrays, they demonstrated a modest but significant downregulation of mRNA levels of genes that were enriched for the miRNA seed sequence. This study and others that followed contributed to the overall view that miRNAs exert silencing through both mechanisms simultaneously, but the more major effect was expected at the protein level, rather than at the mRNA levels.

Recent studies used high throughput proteomics in order to both identify translationally inhibited targets and to more accurately assess the extent of inhibition that a miRNA exerts on mRNA levels and on protein levels [55,56]. These studies reported that individual miRNAs affect hundreds of proteins in the human and mouse out of thousands that were examined. However, the levels of these proteins were decreased only to a relatively mild extent. miRNAs were often before considered as modulators of expression, and their generally observed mild effect on protein levels (and mRNA levels as well) promoted their suggested role as buffers for noise in protein expression, which may confer robustness to developmental programs [57].

Overall, there seems to be a discrepancy between the observation that miRNAs have such subtle effects on protein levels and the fact that their effects on cell fate are so profound. We would like to suggest here one possible model that might bridge this conceptual gap.

## Coupling transcriptional and post-transcriptional miRNA regulation in the control of cell fate

One trivial way to resolve the above discrepancy might argue that the multiplicity of miRNA targets and the simultaneous down-regulation of many proteins might have a cumulative effect, eventually exerting a significant impact on cell fate, even though individual proteins are repressed to a very modest extent. This is a valid argument, particularly since some miRNAs were predicted and shown to have multiple targets within the same pathway [58-60], thus potentially having greater effects on entire pathways than on individual proteins.

**Figure 2.**
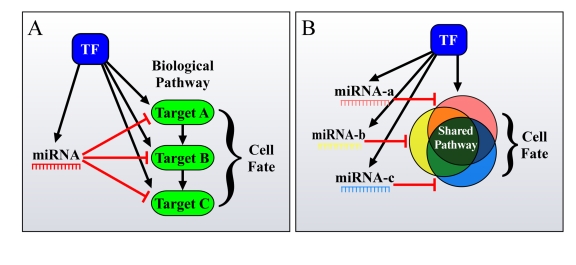
Different ways by which FFLs can account for the enhanced phenotypic effect of miRNAs on cell fate. (**A**) miRNAs and TFs in FFLs tend to mutually target genes from the same pathway. (**B**) Additionally, co-regulated miRNAs and miRNA families co-target many genes in the same pathway, thus resulting in a significant total output, having a major effect on cell fate.

While miRNAs may exert modest effects, yet on many targets, another possible answer to their significant effect on cell fate may lie in the level of the regulatory networks that miRNAs take central part in. miRNAs do not act in isolation, but rather they regulate target genes combinatorially with one another, and are often embedded within intricate regulatory networks together with TFs (Figure 2). In fact, it was demonstrated that at the network level, there is tight coupling between posttranscriptional regulation by miRNAs and the regulation of transcription by TFs [61,62]. Examination of regulatory networks showed that in many cases the same TF controls the transcription of both a miRNA and the targets of that miRNA, or is regulated by the same miRNA with which it shares common targets, forming a diversity of combined transcriptional/post-transcriptional Feed-Forward Loops (FFLs). Collectively, such FFLs potentially regulate thousands of target genes.

**Figure 3.**
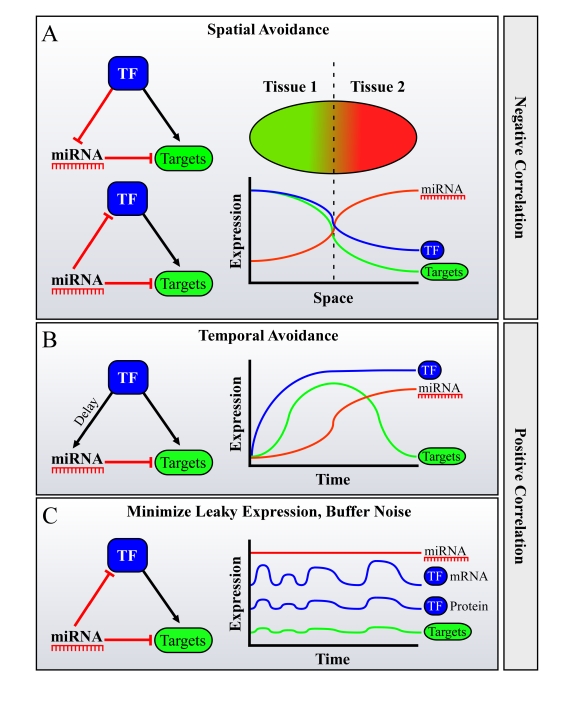
Possible roles for FFLs of miRNAs, Transcription Factors (TFs) and their mutual targets in facilitating spatiotemporal avoidance, or noise buffering. miRNAs are often embedded in Feed-Forward loops (FFLs) with TFs, sharing mutual targets. It was shown that in many cases during development, miRNAs and their targets avoid expression in the same tissue or at the same developmental stage. This phenome-non was termed "miRNA-target spatiotemporal avoidance". The figure depicts how the network wiring of miRNAs in combined transcriptional/posttranscriptional FFLs may explain the spatio-temporal avoidance phenomenon. Different scenarios may facilitate spatial and temporal avoidance, where the TF and the miRNA are either negatively correlated in their expression across tissues (in **A**) or positively correlated, namely are expressed in the same tissue (**B** or **C**). (**A**) Spatial avoidance may be facilitated by the presented FFLs when expression of a miRNA and of a TF anti-correlates across tissues. (**B**) Temporal avoidance may be facilitated by the presented FFL when a miRNA and a TF are co-expressed in the same tissues, creating a temporal shut-down mechanism for their mutual targets, when there is a delay between the activation of the targets by the TF, and its activation of the miRNA. This delay may be achieved for example by a lower affinity binding site of the TF to the miRNA's promoter, by a natural miRNA processing time, etc. (**C**) Buffering of noise in expression may also be facilitated by a FFL wiring when a miRNA and a TF are co-expressed in the same tissues.

Network analyses showed that these FFLs constitute over-represented architectures in the mammalian regulatory network [61,62]. Network FFLs, initially described by Alon and colleagues, were shown to comprise a major component of the transcription networks in bacteria and yeast [63,64]. The discovery that miRNAs and TFs also constitute FFLs offered new possibilities for potential functions for these regulatory units. Clues for the existence of coupling between transcription and miRNA regulation emerged from a very intriguing concept, called miRNA-target avoidance. Two parallel studies, one in *Drosophila* and the other in mammals, showed that during development as well as in adult tissues, miRNA targets often avoid being expressed in the same tissue, or at the same developmental time, as their potential inhibitory miRNA [65,66]. In *Drosophila*, it was shown for some cases that a miRNA and its targets are expressed in adjacent tissues during development, or in consecutive developmental stages, and that miRNAs serve as key players in the precise definition of spatiotemporal differentiation boundaries [66]. This phenomenon was observed also in adult tissues and organs in both *Drosophila *[66] and mouse [65]. Moreover, both studies indicated that this mutual exclusion of miRNAs and their targets does not stem from target degradation by the miRNA. From these two studies, it became evident that posttranscriptional regulation by miRNAs is somehow coordinated with transcription. However, it was not shown originally how, at the mechanistic level, such "miRNA-target spatiotemporal avoidance" is achieved. Combined transcriptional/posttranscriptional FFLs, where the same TF regulates the transcription of both a miRNA and its target genes, or where the miRNA targets a TF and its target genes as well, could serve just that purpose (Figure 3). Such FFLs are thus suggested as a simple mechanism that might facilitate the miRNA-target avoidance phenomenon, where a TF that activates the target genes also represses the miRNA transcription in the tissues in which it is expressed, or the miRNA represses both the TF and its target genes, thereby indirectly causing reduced transcription of its targets in the tissue where it is expressed (Figure 3) [61]. In addition, such FFLs were further suggested to enable the "canalization" and the maintenance of fidelity of developmental processes in general [57].

More recently, evidence has been accumulating that such combined transcriptional post-transcriptional FFLs indeed act as functional units in the regulation of cell fate in many cell types and systems [48,58,67-71]. One striking example, recently published by Marson et al. [69], demonstrated that miRNAs and TFs are involved together in FFLs controlling the maintenance of mouse embryonic stem (ES) cell identity. Consistent with the studies mentioned above [2,3,8,11], which showed that complete miRNA ablation from ES cells eliminates their differentiation capacity, Marson et al. showed that several FFLs involving miRNAs and ES cell TFs act to regulate ES cell identity and differentiation. For example, the *miR-290-295 *polycistronic cluster, containing the most abundantly expressed miRNAs in mouse ES cells, is positively regulated by the ES cell TF *Oct4*, whereas its promoter is co-occupied by *Oct4*, *Sox2*, and *Nanog*. In addition, *miR-290-295 *co-regulate mutual target genes along with these same TFs. Intriguingly, while *miR-290-295* is a rodent specific cluster, a similar FFL involving *Sox* and *Oct4* was computationally predicted in humans [61]. This FFL comprises *miR-302*, which shares the same seed as the rodent-specific *miR-290-295*, and was shown to be highly expressed in human ES cells [72], perhaps serving as a *miR-290-295* human ortholog. Consideration of these results in the perspective of previous studies on miRNAs role in ES cell differentiation supports the conjecture that miRNA-involving FFLs might play an important role in this context, and suggest potential conserved roles for similar FFLs in the maintenance of human ES cell identity as well.

A different perspective on miRNA-TF FFLs was recently provided by Brosh et al. [58]. In this study, a family of 15 homologous miRNAs transcribed as three polycistrons: miR-106b/93/-25, miR-17-92 and miR-106a-363, were shown to form a proliferation-promoting FFL together with the transcription factor E2F. These miRNAs were shown to target a whole battery of anti-proliferative E2F target genes. Most importantly, the study demonstrated that in normal fibroblasts p53 inhibits this FFL as a central step towards cellular senescence. When this inhibition is perturbed by overexpression of the miRNAs, normal cell fate is altered; proliferation is accelerated and senescence is delayed. In agreement with these results, breast cancer tumors bearing mutated p53 showed an elevation in the levels of these miRNAs and were characterized by a high tumor grade, hinting at the role of these miRNAs in promoting proliferation and aggressiveness also in vivo in tumors. This miRNA family was indeed reported in several independent studies to be related to promotion of cancer [58,73,74] (also reviewed in [75]). The above study illustrates how deregulation of the entire FFL may contribute to aberrant proliferation. It also reveals another concept of network wiring of miRNAs, namely combinatorial regulation, and more specifically combinatorial regulation by family-related miRNAs (Figure 2). Combinatorial regulation by miRNAs was globally predicted based on co-occurrence of miRNA target sites in common gene sets [61], and was also observed experimentally [58,76].

miRNAs can be grouped by mature sequence similarity into miRNA families. In some cases, as in the case of the *miR-106b/93/-25* family mentioned above, these families are shown to represent paralogous groups of miRNAs of a common evolutionary origin [77]. Just as paralogous genes were duplicated during evolution but retained some degree of sequence similarity, these paralogous miRNAs share similarity in their sequence, which immediately suggests that they might also share common target genes. More intriguingly, it seems that in many cases such families had not only retained similar targets, but also retained similar transcriptional programs. As described by Brosh et al. [58], the above family of 15 miRNAs retained their joint transcriptional regulation by *E2F*. Coordinated transcriptional regulation of a family of miRNAs, sharing similar targets, all of which are part of the same pathway (in this case negative regulators of proliferation), may have a cumulative effect on the overall levels of proteins in the pathway, thus resulting in a strong effect on cell fate.

Coordinated regulation of family miRNAs was also shown in other cases [78,79]. For example the *miR-34* family, consisting of two transcription units and three mature family members, were all shown to be transcriptionally activated by *p53* and to contribute to apoptosis [80,81], G1 cell-cycle arrest [82] and senescence [83]. Moreover, *miR-34a* and *miR-34c* were shown to target *c-MYC* [46, 84]. In addition, in both mouse and human ES cells, several related miRNA families, often sharing similar seeds, were shown to be co-expressed [69,72]. Moreover, miRNAs from the same family were indeed verified experimentally to have many shared targets [76].

Overall it seems that combinatorial regulation of miRNAs, particularly from the same family, and shared transcription programs for such miRNAs and their common targets portray intricate network architecture (Figure 2). Such architecture is not only over-represented [61], but may also cumulatively generate a strong output that is likely to account for the observed effects on cell fate, and for its alteration when the miRNAs are mis-regulated.

## Concluding Remarks

It is intriguing that despite a relatively mild influence of individual miRNAs on protein levels they are indispensable to various cellular and organismal processes, including control of cell fate and maintenance of genomic integrity. One possible explanation for this may lie in the level of regulatory networks in which miRNAs are embedded. Indeed, joint miRNA-TF FFLs are not only an over-represented architecture in the network but a recurring principle of miRNA regulation of cell fate.

The connection between cell fate and the wiring of miRNAs in coupled transcription/post-transcriptional networks is appealing, and the multiple evidence outlines here serve to support it.

Two principles are common to the different examples discussed above: 1. miRNAs are embedded in combined transcription-nal/post-transcriptional FFLs that co-target many genes.

2. Several co-regulated miRNAs act together to exert their regulation on target genes involved in the same pathway.

However, more studies should be undertaken in order to fully establish the link between the network wiring of miRNAs in transcriptional/post-transcriptional FFLs and their effect on cell fate. A recent study demonstrated that the wiring of *miR-7* in a network of FFLs in the fly equips the network with robustness to environmental perturbation [68]. Such approach suggests that when studying possible roles for miRNAs, one should consider them as parts of a larger regulatory network, rather than adopting the reductionist view of single miRNA - single target. Our recognition of the centrality of miRNAs in the regulatory network may help us to elucidate how miRNAs exert such profound impact on cell fate.
